# Effects of Core Training on Sport-Specific Performance of Athletes: A Meta-Analysis of Randomized Controlled Trials

**DOI:** 10.3390/bs13020148

**Published:** 2023-02-09

**Authors:** Kuan Dong, Tenghao Yu, Buongo Chun

**Affiliations:** Graduate School of Physical Education, Myongji University, Yongin 17058, Republic of Korea

**Keywords:** athletes, player, core training, sport-specific performance, meta-analysis

## Abstract

Improving athletes’ performance is a major topic of interest in studies on competitive sports. Core training has been used as a training method in daily life and rehabilitation, and recently, in competitive sports, with positive results. Previous experimental studies showed that core training can improve the fitness level of athletes (e.g., balance, core stability, etc.), but offer no consistent conclusions on whether it can improve sport-specific performance. The purpose of this study was to investigate the effect of core training on the sport-specific performance of athletes through a meta-analysis. Relevant studies on randomized controlled trials were selected, and we calculated the effect size using standardized mean difference values and the random effects model. Results showed that core training had almost no effect on athletes’ power and speed, while agility showed a medium effect size but no statistical significance. On the other hand, there was a large effect on general athletic performance, such as core endurance and balance. Consequently, core training had a great effect on the core endurance and balance of athletes, but little effect on sport-specific performance. This result implies that more elaborate core training programs should be designed to improve the sport-specific performance of athletes.

## 1. Introduction

A major topic of interest in sport events is the development of effective training methods to improve the performance of athletes. While some previous studies have proven the effect and development of various training interventions, such as speed, agility, and quickness (SAQ) training [1,2], weight training [3,4], plyometric training [5,6], and complex training [7,8,9], some others focused on systematic studies and meta-analyses of different training methods [10,11,12].

Core training, which has been receiving attention recently, has been reported to have positive effects on daily life and rehabilitation [13,14,15]. The core is a unit formed by the waist, pelvis, and hip. As the intermediate ring of the human body, the term specifically refers to the area below the shoulder joint, including the pelvis, and above the hip [16]. Two types of muscle fibers compose the core muscles: slow-twitch and fast-twitch. Slow-twitch fibers primarily make up the local muscle system (the deep muscle layer). Key local muscles include transversus abdominus, multifidi, internal oblique, deep transvers spinalis, and the pelvic floor muscles. Fast-twitch fibers make up the global muscle system (the superficial muscle layer). Key global muscles include erector spinae, external oblique, rectus abdominis muscles, and quadratus lumborum [17]. The core is particularly important in sports because it provides “proximal stability for distal mobility” [18].

The core muscle is a key factor that stabilizes the spine and trunk during exercise, while maximizing leg balance and athletic performance. A strong core muscle not only makes the body more efficient, but also plays a key role in performing an integrated motor function that delivers the force generated in the trunk and pelvis to the limbs. It can also improve physical balance and develop neural control, the functions of muscles, coordination skills, proprioception, and other types of muscular strength [18,19]. Moreover, the enhancement of the “Lower Pelvic Unit” in the core muscles led to a better realization of “core control,” and the core muscles had a better effect on the prevention of groin pain and the treatment of lower back pain [20,21,22]. For this reason, the core muscle has become one of the main focal points in training athletes. Several randomized controlled trials (RCTs) related to core training have been published, and there is increasing experimental evidence that core training improves the fitness level and sport-specific performance of athletes [23,24,25,26].

While studies demonstrated the positive effect of core training on the physical strength of athletes, they show different results regarding the effect on sport-specific performance. Kuhn et al. [27] reported that core training improved core endurance and maximal isometric strength in handball players; however, it did not improve the throwing speed, which is important in performance. In soccer players, Murat et al. [28] showed that core training can improve agility but not speed. Ozmen and Aydogmus [29] found that core training could improve balance and core endurance in badminton players, but not agility. This may be because abilities such as agility take longer to show significant improvement [29]. In the last few years, several experiments have investigated the effect of core training on athletic performance; however, results were inconsistent and inconclusive. At the same time, most systematic studies and meta-analyses focused on the effects of core training on injuries and diseases [30,31], while to our knowledge, no studies considered its effects on the sport-specific performance of athletes. Thus, a comprehensive summary and quantitative analysis of this phenomenon are needed, which is the purpose of this systematic review and meta-analysis. As such, this study evaluated the effect of core training on athletic performance (e.g., exercise performance, agility, endurance, and balance) of athletes, concluding that core training greatly affects the core endurance and balance of athletes, while it has little effect on sport-specific performance.

## 2. Materials and Methods

This study performed a systematic literature review and meta-analysis on the effects of core training on the athletic performance and sport-specific athletic performance of athletes. We conducted the study according to the systematic review flowchart presented in preferred reporting items for systematic reviews and meta-analysis (PRISMA). Our research program has been registered on Prospero, the International Register of Expectations for System Evaluation, with the registration number CRD42022371302.

### 2.1. Search Strategy

Regarding the search for the literature review, electronic databases such as EBSCO, PubMed, and Cochrane were used. We combined keywords such as “((athlete) OR (player)) AND ((((core training) OR (core exercise)) OR (core stability) OR (core strength training) AND (randomized controlled trial)).” The data search was conducted independently by two researchers, and the article reference list was cross-checked to supplement the search.

### 2.2. Inclusion and Exclusion Criteria

#### 2.2.1. Inclusion Criteria

In the selection of studies for the literature review, the following criteria had to be met for inclusion: (1) Studies on athletes; (2) Studies using only core training as an intervention; (3) Studies in which the control group did not perform alternative or general training; (4) Studies in which at least one indicator out of general athletic performance (core endurance and balance) or sport-specific performance (speed, power and agility) was included in their results; and (5) Studies in which the study design was a randomized control group experiment.

#### 2.2.2. Exclusion Criteria

The exclusion criteria for the literature review were: (1) Studies that were not randomized controlled trials; (2) Studies in which the data of the experimental and control groups did not follow the same baseline; (3) Studies not providing clear data on statistical values, such as means and standard deviations; and (4) Studies whose full text was not found.

### 2.3. Research Option

**Figure 1:**
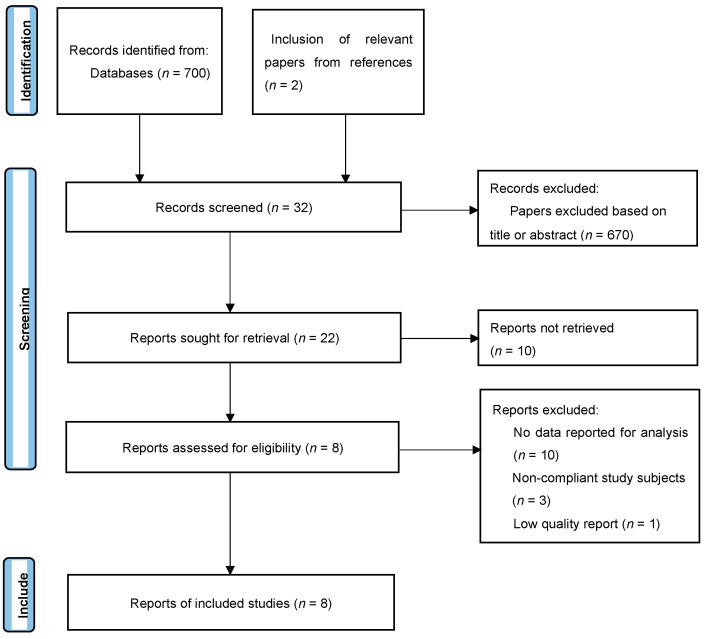
PRISMA flow chart (2020) for the inclusion and exclusion of studies.

Two researchers read the titles and abstracts, deciding on a preliminary inclusion or exclusion of studies. Duplicate studies were identified from those included, and the final decision to include a study was made after reading the full texts. In the case of disagreement on the selection and exclusion of studies, the decision was made by discussing with a third-party researcher (Figure 1).

### 2.4. Data Extraction

The following data were extracted from the selected literature and recorded: (1) Author; (2) Sample size; (3) Age; (4) Sport; (5) Test indices; (6) Duration, frequency, and amount of training; (7) Intervention measures; (8) Experimental results; and (9) Competitive level: professionals (the first division of their countries or above, Greek Super League, Champions League, Norwegian Premier League, Brazilian First Division, Morocco First Division, Swedish First Division, Ireland First Division, etc.) and under level: semi-professional [32].

### 2.5. Risk of Bias Assessment on Individual Studies

The risk of bias (ROB) was assessed using the Cochrane Handbook Version 5.1.0 for the ROB assessment in RCT, through the following 7 items: (1) Random sequence generation; (2) Allocation concealment; (3) Blinding of participants; (4) Blinding of outcome assessment; (5) Incomplete outcome data; (6) Selective reporting; and (7) Other bias. ROB is divided into three levels according to the number of components that can have high ROB in the experiment: high risk (5 or more), medium risk (3 or 4), and low risk (2 or less) [33].

### 2.6. Statistical Analysis

The meta-analysis of this study was conducted using the Review Manager software (RevMan 5.4, Cochrane Collaboration, Oxford, UK). The effect size was calculated using the mean and standard deviation for the outcome variables. The inverse variance random effects model was used to indicate the effect sizes as standardized mean difference (SMD) and 95% confidence interval (CI). The calculated effect sizes were interpreted using the rules outlined by Cohen, Hopkins et al. [34] for SMD. Statistical heterogeneity was measured using the I^2^ test (I^2^ ≤ 25% classified as low heterogeneity, 25% < I^2^ < 75% as moderate heterogeneity, and I^2^ ≥ 75% as high heterogeneity) [35]. To verify studies’ publication bias, a funnel plot representing the relationship between sample size and effect size was used.

## 3. Results

### 3.1. Literature Search and Flowchart

We selected 700 articles through an initial search on the database. In the first round of screening, we read the titles and abstracts and excluded 670 articles not related to the literature review and the research topic. In the second round of screening, we additionally excluded studies in which the full text was not available (*n* = 10), studies with ineligible subjects (*n* = 3), studies not clearly providing outcome data (*n* = 10), and studies of poor quality (*n* = 1). From the references of previous studies, we selected two additional articles that met our selection criteria. Finally, eight articles that met all inclusion criteria were included in the analysis (Figure 1).

### 3.2. Characteristics of Study Participants

Table 1 and Table 2 show the characteristics of the eight selected studies [29,36,37,38,39,40,41,42]. This review included 169 athletes participating in seven sport events (86 in the experimental group and 83 in the control group). The participants were mostly male athletes (male, *n* = 116; female, *n* = 9; N/A, *n* = 44), and the age of the subjects varied from 11 to 21. For exercise intervention, the experimental group performed core training, while the control group performed traditional strength training or no training at all. The training period for all included RCT studies was between 4 and 12 weeks. Core training was performed 20–40 min per session, and the frequency of exercise was 2–4 times a week.

### 3.3. Quality Assessment

**Figure 2:**
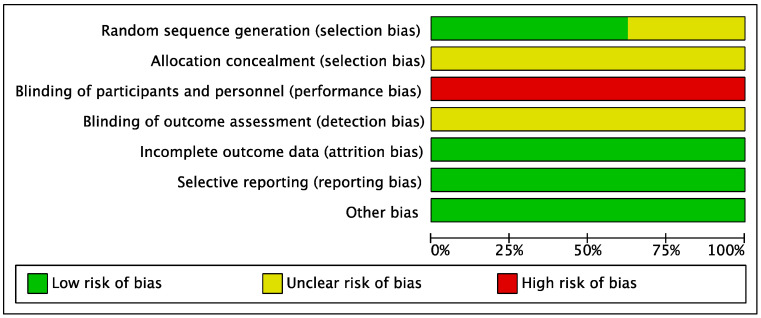
Risk of bias graph.

**Figure 3:**
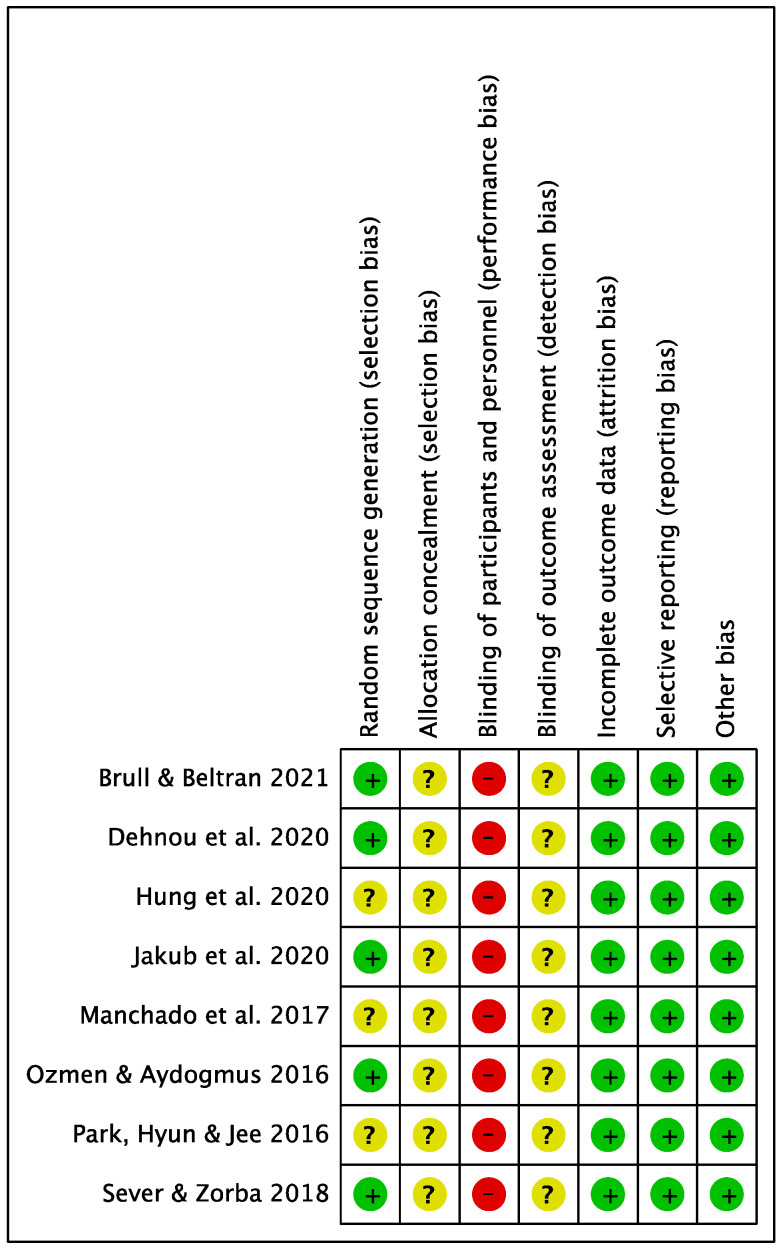
Risk of bias summary. (Green: low risk; Yellow: unclear risk; Red: high risk) [29,36,37,38,39,40,41,42].

The results of assessing the ROB are shown in Figure 2 and Figure 3. Six studies described randomization methods, and two studies only mentioned randomization without providing detailed explanations. None of the studies explained specific methods related to allocation concealment. Due to the nature of core training, all studies were considered to have a high risk of performance bias. The risk of attrition bias and reporting bias was low in all seven RCTs. Therefore, all other types of bias, except for performance bias, were low in the examined studies.

### 3.4. Publication Bias Assessment

**Figure 4:**
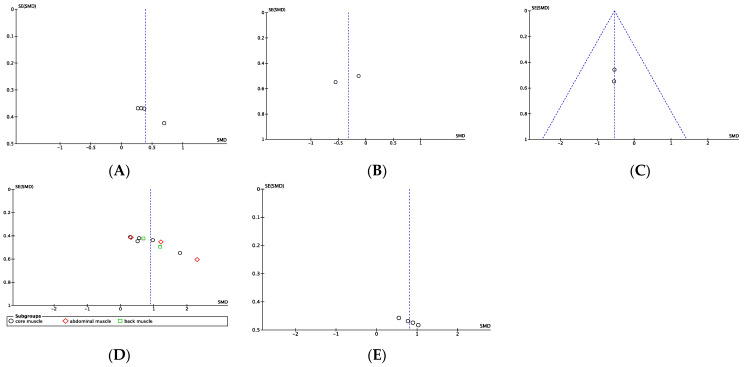
Funnel plot. (**A**) Sport-specific power; (**B**) Sport-specific speed; (**C**) Sport-specific agility; (**D**) Endurance; (**E**) Balance.

As shown in the funnel plot (Figure 4), speed and balance (Figure 4B and Figure 4E, respectively) are evenly distributed on both sides of the SMD and are located at the top of the funnel plot, indicating that there is no publication bias in the study. Figure 4C,D showed potential publication bias.

### 3.5. Meta-Analysis Results

#### 3.5.1. Sport-Specific Performance

**Figure 5:**
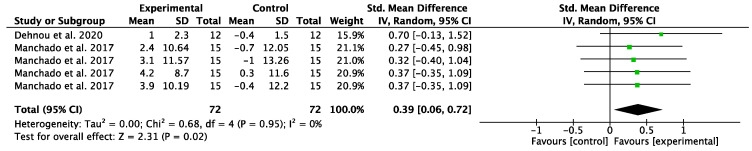
The effects of core training on the power performance of athletes (e.g., the effect on wrestlers’ medicine ball throwing speed [m/s]). CI = confidence interval, df = degrees of freedom, IV = inverse variance, random effects model, SE = standard error, SMD = standardized mean difference [38,39].

**Sport-specific power.** The results of the meta-analysis determining core training effectiveness on the sport-specific performance of athletes showed that core training generated a small effect size in power compared to the control group (SMD = 0.39; 95% CI: 0.06–0.72; I^2^ = 0%; *p* = 0.02); see Figure 5.

**Figure 6:**
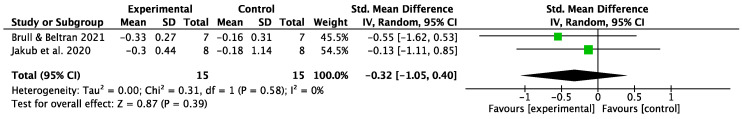
The effects of core training on the speed of athletes (e.g., swimming 50 m speed). CI = confidence interval, df = degrees of freedom, IV = inverse variance random effects model, SE = standard error, SMD = standardized mean difference [36,40].

**Sport-specific speed.** Figure 6 displays the results of the meta-analysis determining the effectiveness of core training on athletes’ time trial, as a sport-specific athletic performance. It shows that core training had a small effect size in specific athletic performance (time trial) compared to the control group (SMD = −0.32; 95% CI: −1.05–0.40; I^2^ = 0%; *p* = 0.39).

**Figure 7:**
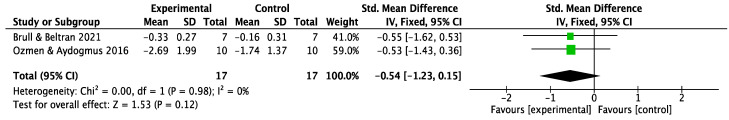
The effects of core training on the agility of athletes (e.g., Illinois Agility Test). CI = confidence interval, df = degrees of freedom, IV = inverse variance, random effects model, SE = standard error, SMD = standardized mean difference [29,36].

**Sport-specific agility.** We conducted a meta-analysis to determine the effectiveness of core training on the athletic performance of athletes (see Figure 7). The results showed that core training determined a medium effect size in agility (e.g., Illinois agility test) compared to the control group (SMD = −0.54; 95% CI: −1.23–0.15; I^2^ = 0%; *p* = 0.12).

#### 3.5.2. Core Endurance

**Figure 8:**
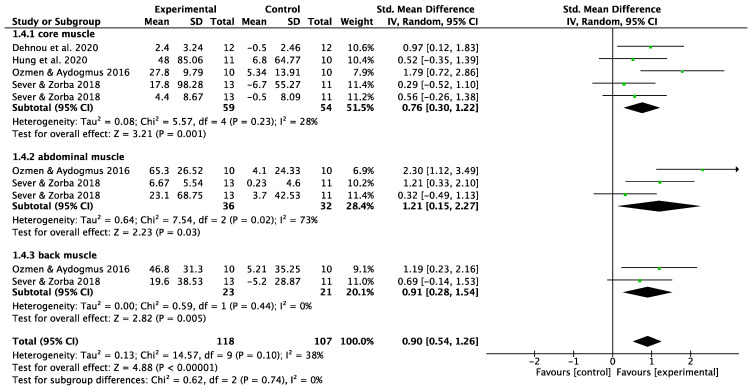
The effects of core training on athletes’ core endurance (e.g., time in plank test). CI = confidence interval, df = degrees of freedom, IV = inverse variance, random effects model, SE = standard error, SMD = standardized mean difference [29,37,39,41].

The meta-analysis results in Figure 8 showed that core training had a large effect size in core endurance compared to the control group (SMD = 0.90; 95% CI: 0.54–1.26; I^2^ = 38%; *p* < 0.00001). A subgroup analysis showed a medium effect size in anterior abdominal endurance (plank time; SMD = 0.76, 95% CI: 0.30–1.22, I^2^ = 28%, *p* = 0.001), and a large effect size in lateral abdominal endurance (side plank time; SMD = 1.21, 95% CI: 0.15–2.27, I^2^ = 73%, *p* = 0.03) and posterior back endurance (bridge time; SMD = 0.91; 95% CI: 0.28–1.54; I^2^ = 0%; *p* = 0.005). A high level of heterogeneity was found in the lateral abdominal muscles. To resolve this, a sensitivity analysis was conducted from which the data provided by Ozmen and Aydogmus [29], which was presumed to be the main cause of heterogeneity, was excluded. Consequently, the statistical significance changed (SMD = 0.75; 95% CI: −0.12–1.62; I^2^ = 53%; *p* = 0.09).

#### 3.5.3. Balance

**Figure 9:**
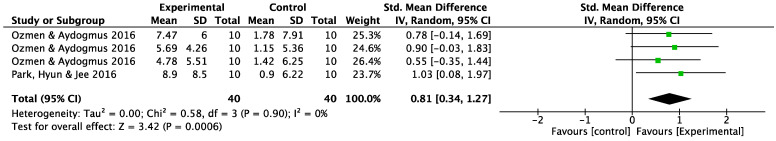
The effects of core training on the balance of athletes (e.g., star excursion balance test). CI = confidence interval, df = degrees of freedom, IV = inverse variance, random effects model, SE = standard error, SMD = standardized mean difference [29,42].

The results in Figure 9 show that core training had a large effect size on balance, as opposed to the control group (SMD = 0.81; 95% CI: 0.34–1.27; I^2^ = 0%; *p* = 0.0006).

## 4. Discussion

This study was conducted as a systematic literature review and meta-analysis to investigate the effects of core training on physical strength and the sport-specific athletic performance of athletes. To this end, eight randomized controlled trials were included in the meta-analysis, based on which we explored how core training affected 169 athletes. The results showed that core training had a significantly large effect on the physical strength of athletes (core endurance and balance) compared to the control group and had a small effect on sport-specific athletic performance (speed, agility, and power). As far as we know, this study is the first to analyze the effects of core training on the sport-specific athletic performance of athletes. Its results emphasize that it is necessary to more elaborately design core training programs to improve the performance of athletes. Unfortunately, although these theories are relatively well known, the contents of core training in the field do not include sport-specific movements [24,29,38]. Therefore, it is thought that a core training program reflecting the characteristics of the event should be performed in the field with reference to this comprehensive result.

Our meta-analysis showed that core training could improve the sport-specific power, speed, and agility of athletes to some extent, but the effect was small or medium [27,38]. Behm et al. [43] proposed the concept of specificity in training and claimed that training must mimic sport-specific motions and meet certain requirements of sports. The studies selected showed that most training programs are carried out through isometric contraction in a horizontal position (e.g., plank and bridge) and through explosive isotonic contraction exercises in an upright position for sport events (e.g., running and throwing). Moreover, training in unstable conditions may reduce power output [44,45], muscular strength, or speed [37]. Furthermore, it has been suggested that restriction of the kinetic chain to the fascia may inhibit muscle function and affect movement patterns [46]. This may be the reason why core training had a slight effect on sport-specific performance. Meanwhile, the core training intervention carried out in Manchado et al.’s study [38] showed a positive effect on sport-specific performance. This training program had three levels of gradually increased difficulty, consisted of seven exercises for each core muscle, developed muscle endurance and muscular strength through static and dynamic training, and performed plane motion and multi-joint exercises of closed kinetic chain.

Results further showed that core training had a large effect on the general athletic performance of athletes, such as core endurance and balance [47]. Van Pletzen et al. [48] discovered a positive correlation between the static muscle endurance of core trunk muscles (e.g., Bunkie test) and the physical ability of rugby players through an experimental study. Core training improves neuromuscular coordination in the core area, increases muscular strength in the lumbar–pelvis area, and increases limb stability and energy transfer during exercise [18]. At the same time, proximal core activation improves the distal function efficiency, allowing multi-joint muscles to work more ably to control spinal movement [49], thereby affecting athletes’ activation of muscles in a more coordinated way or generate more power [50]. This can help increase athletes’ fitness level. Meanwhile, the abdominal muscle showed heterogeneity in core muscle endurance. The sensitivity analysis identified Ozmen and Aydogmus ’s [29] study as the cause. The subjects in this study were athletes around the age of 11, and core training performed during the period of rapid physical development leads to a great improvement effect. Moreover, the study conducted by Sever et al. [37] performed only dynamic core training, whereas Ozmen and Aydogmus [29] obtained better results by combining static and dynamic core training, which explains the heterogeneity. Furthermore, considering that an increase in muscle activation is induced by the verbal instructions of the coach in resistance training, it seems that using active verbal instructions in training may also be necessary for maximizing the training effect [51]. In addition, the difference between male and female pelvic morphology leads to greater deviation of the buttocks and torso in female exercise. Thus, it can be assumed that female athletes benefit more from core training than male athletes [52].

### Study Limitations

The included randomized controlled trials had a relatively high publication bias, thereby affecting the reliability of the systematic review, as well as the meaning of the results. For future experiments on core training, researchers must use suitable randomization and allocation concealment, blinding, and statistical methods to reduce the ROB of the study design. Therefore, we encourage the participation of experts in biostatistics and evidence-based medicine. Among the included studies, three were about soccer. The rest of the sports studied had only one study each, which would also influence the results to some extent. In the future, studies verifying the sport-specific performance effect of core training in various sports should be added.

## 5. Conclusions

While core training had a good effect on general athletic performance, such as the core muscle endurance and balance of athletes, it had lower effect on sport-specific athletic performance. These results suggest that it is necessary to adequately design core training programs to improve sport-specific athletic performance. In particular, adding core training movements suitable for the characteristics of the sport event will help improve the athletes’ performance. In addition, the methodological quality of the reviewed studies turned out to be low, which suggests the need to perform more strictly designed RCTs in the future.

## Figures and Tables

**Table 1:** Characteristics of study participants.

Study	Sport	Competitive Level	Study Design	No. of Subjects			Age (Mean ± SD)	Performance Tests	Duration, Frequency	Exercise Interventions	Outcome
				Total (M, F)	EG	CG					
Brull and Beltran 2021 [36]	Soccer	semi- professional	RCT	14 (14,0)	7	7	17.1 ± 0.69	10 m sprint, 25 m VC	8 weeks, 2 sessions/week, 20 min/session	Experimental group: unilateral skater squat with elastic band, unilateral linear sprint with elastic band, etc. Repeated 10 times, 10 s rest. Control group: traditional training.	10 m Sprint ↑, *p* > 0.05; 25 m VC ↑, *p* > 0.05
Sever and Zorba 2018 [37]	Soccer	semi- professional	RCT	24	13	11	17.7 ± 1.3	Core stability (leg raise, push-up, plank crunch, back isometric)	8 weeks, 3 sessions/week, 30 min/session	Experimental group: crunch, Russian twist. 20–35 × 2, etc., incremental training. Control group: no intervention.	↑, Core stability (leg raise *p* > 0.05); Crunch, Back isometric, *p* < 0.01; Plank, Crunch, *p* < 0.05
Ozmen and Aydogmus 2016 [29]	Badminton	semi- professional	RCT	20 (11,9)	10	10	10.8 ± 0.3	SEBT, core endurance, IAT	6 weeks, 2 sessions/week	Dynamic 20 s, static exercises 20 reps, lunge with medicine ball twist, abdominal bracing, hollowing, pelvic bridge alternating knee extension and shoulder, etc. Control group: no intervention.	SEBT ↑, *p* < 0.05 Core endurance ↑, *p* < 0.05 Agility ↑, *p* > 0.05
Manchado et al. 2017 [38]	Handball	semi- professional	RCT	30 (30.0)	15	15	18.7 ± 3.8	Throwing velocity	10 weeks, 4 sessions/week, 20–25 min/session	Experimental group: 3 stages, gradually increase the difficulty, crunch or curl-up with Swiss ball, frontal bridge with Swiss ball, etc. 228 and 238 ECOs. Control group: no intervention.	↑, *p* < 0.05
Dehnou et al. 2020 [39]	Wrestling	semi- professional	RCT	24 (24,0)	12	12	16.8 ± 1.1	OMBT, suplexes, bridges, MBCT	4 weeks, 3 sessions/week, 20 min/session	Experimental group: forearm plank, side plank, reverse plank, one-arm standing, dumbbell hold. Repeat 3 times, rest 1–2 min. Control group: no intervention.	OMBT ↑, *p* < 0.05 Suplexes ↑, *p* > 0.05 bridges ↑, *p* < 0.05 MBCT ↑, *p* > 0.05
Jakub et al. 2020 [40]	Swimming	professional	RCT	16 (16,0)	8	8	20.2 ± 1.17	50 m swimming speed	6 weeks, 3 sessions/week, 25 min/session	Three stages, flutter kicks, single-leg V-ups, prone physio ball trunk extension, Russian twists, etc. Duration 40 s, rest 25 s. Control group: no intervention.	T swimming speed ↑, *p* < 0.05
Hung et al. 2020 [41]	Soccer, basketball, etc.	semi- professional	RCT	21 (21,0)	11	10	NA	SEPT	8 weeks, 3 sessions/week, 30 min/session	3 stages, basic strength, stability, functional strength. Four exercises per stage, bridge, side plank, etc. Control group: no intervention.	SEPT ↑, *p* > 0.05
Park, Hyun and Jee 2016 [42]	Archery	semi- professional	RCT	20	10	10	17.3 ± 1.06	Balance	12 weeks, 3 sessions/week, 40 min/session	Experimental group gradually increased the training volume in 3 stages, basic bridge, bridging variation, double-leg stretch, etc. Borg’s scale 11–13. Control group: no intervention.	↑, *p* < 0.05

M: male; F: female; EG: experimental group; CG: control group; IAT: Illinois agility test; RCT: randomized controlled trial; SEBT: star excursion balance test; OMBT: overhead medicine ball throw; MBCT: medicine ball chest throw; SEPT: specific endurance plank test; VC: V-Cut test. ↑: Increased.

**Table 2:** Performance tests.

Performance Test	Procedures
10 m sprint	The fastest possible sprint time was recorded using photoelectric cells (Chronojump BoscoSystem, Barcelona, Spain). The participants started from a static position with one leg forward, according to preference, 1 m before the starting line.
25 m V-Cut	25 m test with 4 changes of direction of 45° every 5 m. The time to sprint the 25 m with the fastest possible direction changes was recorded using photoelectric cells (Chronojump BoscoSystem, Barcelona, Spain).
Core stability	Two were chosen among the isotonic and three were chosen among the isometric type of exercises. While the static tests consisted of leg raise, plank and isometric extension, the dynamic tests included sit-up and push-up tests.
SEBT	The reach directions were determined by affixing three tape measures to the gymnasium floor, one orientated anterior to the apex (A) and two aligned at 135° to this in the posteromedial (PM) and posterolateral (PL) directions. Each subject was instructed to reach as far as possible with the dominant leg in each of the 3 directions while maintaining a single leg stance.
Core endurance	For the side bridge test (SBT), subjects laid on their side with their legs extended on a treatment table, resting on their forearm with the elbow flexed to 90°. Subjects were instructed to lift their hip off the table with the other arm and hand across the chest, resting on the opposite shoulder. For the abdominal fatigue test (AFT), the subjects were seated with their back resting against a wedge that maintained a 45° flexion from the horizontal on the treatment table. Knees were flexed to 90° and the feet were stabilized by a researcher. For the back-extensor test (BET), the subjects laid on the treatment table in a prone position with the upper body cantilevered out over the end of the table. The test was terminated when the body position could no longer be maintained. Time was recorded in seconds using a stopwatch.
IAT	The course was set up on a basketball court and had a length and width of 10 m and 5 m, respectively. Four cones marked the start, finish, and the two turning points. Another four cones were placed down the center in equal intervals. The cones in the center were spaced 3.3 m apart. Their time was measured by photocells located from start to finish, and the best result of the two attempts were recorded.
Throwing velocity	A radar (StalkePro Inc., Plano, TX, USA), with recording frequency of 33 Hz and sensitivity of 0.045 m·s1, was used to measure the upper body’s throwing velocity. The test consisted of throwing from four different positions: (1) From the penalty position (7 m); (2) A standing throw without a run-up from the free-throw line (9 m); (3) A standing throw with a run-up from 9 m; and (4) A jump throw with a run-up from 9 m. In each case, the best of the three attempts was recorded for further analysis.
OMBT	Overhead medicine ball throw (OMBT), using a 5 kg medicine ball; medicine ball chest throw (MBCT), using the same medicine ball as OBMT; the maximum number of suplexes performed in 30 s, and maximum number of Bridges completed in 30 s. The OMBT that was conducted consisted of downward and upward phases of movement. In the starting position, the participants stood upright with their feet shoulder-width apart and heels aligned at the starting line whilst carrying the medicine ball. During the downward phase of the movement, the participants slightly flexed at the knees and trunk whilst positioning the medicine ball between their lower limbs. During the upward phase, the participants undertook a triple extension maneuver through their ankles, knees and hips whilst thrusting the medicine ball overhead. For the MBCT, the participants sat on a chair and threw the medicine ball using a chest press maneuver. The athletes were allowed three attempts for both the OMBT and MBCT, with approximately 1 min of rest in-between, and the greatest distance was recorded.
Suplexes	
Bridges	
MBCT	
50 m swimming	The total time needed to cover the distance of 50 m from the starting signal until the wall is touched by the hand of the swimmer at the end.
Balance	Humac Norm Balance System (Computer Sports Medicine Inc., Boston, MA, USA)

V-Cut: change-of-direction maneuverability test.

## Data Availability

The data that support the findings of this study are available from the corresponding author (B.C.) upon reasonable request.
